# Randomized Clinical Trial to Evaluate the Efficacy and Tolerability of Nebulized Hyaluronic Acid and Xylitol Based Solution after Septoturbinoplasty

**DOI:** 10.3390/jpm13071160

**Published:** 2023-07-20

**Authors:** Peter Baptista, Antonio Moffa, Lucrezia Giorgi, Manuele Casale

**Affiliations:** 1 Department of Otorhinolaryngology, Clinica Universidad de Navarra, 31008 Pamplona, Navarra, Spain; 2 ENT Department, Al Zahra Private Hospital Dubai, Dubai 23614, United Arab Emirates; 3 Integrated Therapies in Otolaryngology, Fondazione Policlinico Universitario Campus Bio-Medico, 00128 Rome, Italy; l.giorgi@unicampus.it (L.G.); m.casale@policlinicocampus.it (M.C.); 4 School of Medicine, Università Campus Bio-Medico di Roma, 00128 Rome, Italy; 5 Unit of Measurements and Biomedical Instrumentation, Università Campus Bio-Medico di Roma, 00128 Rome, Italy

**Keywords:** Aluneb, hyaluronic acid, xylitol, nasal surgery, septoplasty, turbinate submucosal resection

## Abstract

Septoplasty and turbinate surgery are among the most frequent surgical procedures to improve nasal obstruction and quality of life. These procedures usually imply the presence of congestion, secretions, and crusting related to the movement of the instruments during surgery. However, the use of nasal lavage may reduce this situation. The addition of Hyaluronic acid or Xylitol offers advantages in these washes. This study was a randomized, double-blind, controlled trial. All patients underwent endoscopic septoplasty with inferior turbinate submucosal resection without posterior nasal packing. SNOT-22, main VAS, NOSE, Modified Lund-Kennedy endoscopic scale, number of crusts and adhesions were quantified before and on the day of the surgery, visit three (seven days), visit four (fourteen days), and visit five (twenty-eight days). Forty-seven patients completed the study, divided into a standard saline arm (group 1, 22 patients) and normal saline plus HA and Xylitol arm (group 2, 27 patients). Both treatment groups improved their quality of life and objective parameters during the four weeks of the study. All patients tolerated the nasal irrigations well, and none discontinued the treatments. The study concludes that nasal washes of Aluneb Isotónico^®^ offer several benefits to patients as a protective and preventative agent.

## 1. Introduction

Septoplasty and turbinate surgery are two of the most frequent surgical procedures performed by an ENT to improve nasal obstruction and quality of life. These techniques provide maximum functional and respiratory improvement while preserving the physiologic functions of the nose (filtering, warming, and moisturizing the air) to enhance nasal flow [1]. Moreover, operative recovery usually lasts only a few weeks, and serious complications are rare [2]. These surgical techniques usually imply the presence of congestion, secretions, and crusting related to the movement of the instruments during surgery. The use of nasal lavage may reduce this situation. After endonasal surgery, painless nasal washes are essential to postoperative nasal care and eliminate secretions, crusts, and debris. This procedure is necessary for complete recovery, especially in functional endoscopic sinus surgery (FESS) (Grade 1A strength of recommendation [3]). Several different solutions are usually used for nasal washes: isotonic, hypertonic, or alkaline-buffered saline, as well as seawater, have been used for nasal lavage. Among them, the isotonic saline solution is most frequently used [4]. Recently, several studies have shown that adding certain ingredients to nasal washes, such as hyaluronic acid or xylitol, offers advantages in respiratory tract treatments [5,6,7,8].

Hyaluronic Acid (HA) is a naturally occurring non-sulfated glycosaminoglycan. The physicochemical characteristics of high molecular weight HA (HMW HA) (more than 1000 kDa) have a role in how HA carries out its biological functions when administered through the nose. HMW HA can modify tissue hydration, osmotic balance, and the physical characteristics of the extracellular matrix (ECM) due to its macromolecular size and the physical mechanism of action. Other functions of HMW HA include tissue healing (including activation and moderation of inflammatory responses), promotion of cell proliferation, migration, angiogenesis, re-epithelization via the proliferation of basal keratinocytes, and “remodeling” mucous membranes and with anti-edematous action [9]. HMW HA also augments mucociliary clearance and has been shown to reduce the frequency of acute exacerbations of chronic bronchitis. The modulating effect of HMW HA in wound healing and mucosal regeneration has been shown to be safe, tolerable, and efficacious in clinical trials following sinonasal surgery [6]. Recent studies have supported the role of HA in improving the endoscopic and cytological parameters of chronic rhinosinusitis [10]. Studies also show that animals avoid postoperative stenosis in the nose due to the restorative surface action [11,12,13]. In addition, a recent systemic review and meta-analysis analyzed the effect of HA in FESS. The researchers concluded that there is a lower adhesion rate with improved mucosal healing [14]. However, there is still debate about the most effective postoperative way to administer HA in nasal surgery due to the diversity of preparations (absorbable and non-absorbable dressings and topical preparations such as cream, spray, and nebulized ampules) [15]. 

On the other hand, Xylitol is a natural five-carbon sugar alcohol commonly used as a sweetening agent that has recently shown potential in treating recurrent respiratory infections when used in nasal washes thanks to its innate bactericidal and anti-adhesive effects [16,17,18]. Administered intranasally, it has also been shown to be helpful in the postoperative period following septoplasty and endoscopic sinus surgery. It functions as an effective mucolytic by reducing the viscoelasticity and viscosity of mucus, and thus preventing post-surgical crusting and post-surgical nasal mucosal superinfection [19]. Furthermore, it significantly improved nasal congestion, while in allergy patients, it considerably improved rhinorrhea symptoms [20].

Currently, many types of devices exist for the intranasal administration of nasal rinses. The mucosal atomization device (MAD) is widely used to administer drugs for intranasal application, allowing an efficient administration of Aluneb^®^ solutions throughout the nasal cavity.

This study compares whether Aluneb Isotónico^®^ (Hyaluronic acid +Xylitol) provided a better situation for patients with septoplasty and inferior turbinate submucosa resection with complete sparing of the mucosa in comparison to the use of the isotonic saline solution.

## 2. Materials and Methods

### 2.1. Study Design and Setting

This randomized controlled trial was conducted at the Department of Otorhinolaryngology of Clínica Universidad de Navarra, Pamplona, for 18 months between October 2020 and March 2022.

### 2.2. Ethical Considerations

The study was approved by the Comité de ética de la investigación con medicamentos regional de la Comunidad de Madrid (CEIm-R) and conducted following the ethical standards of the Declaration of Helsinki. All patients signed their informed consent before participating in the study. The trial was registered at the AEMPS (Agencia Española de Medicamentos y Productos Sanitarios) under the trial registration No. 20-0041.

The report has been carried out following the general Structure and Content of Clinical Study Reports from the ICH Harmonised Tripartite Guideline; International Recommendations ICH Topic E6, CPMP/ICH/135/95 of 1 May 1996 [16], European Parliament and Council Guideline 2001/20/EC of 4 April 2001 [17].

The information collected from each patient included in the study and the confidentiality of individual patient data were respected. The inclusion of subjects in the study was voluntary, and the participant’s privacy was safeguarded. Good Clinical Practice guidelines (ICH E6 GCP) and appropriate procedures have continuously been followed to ensure compliance with REGULATION (EU) 2016/679 OF THE EUROPEAN PARLIAMENT AND OF THE COUNCIL of 27 April 2016 [18].

### 2.3. Inclusion Criteria

After signing their informed consent, patients were immediately evaluated to determine if they met the eligibility criteria for participating in this study (screening phase) by revising their medical records. Inclusion criteria are summarized in Table 1. 

### 2.4. Randomization and Allocation Concealment

Once all selection criteria were validated, the patient was randomized to participate in the study.

The participants were randomly assigned into two groups. A simple, balanced randomization (1:1) was carried out, ensuring the random distribution of each treatment group (control group and treatment group). Randomization was performed for each treatment by generating random numbers in a Microsoft Excel (Version 2306 Build 16.0.16529.20164) template designated to perform the function. The randomization codes for each participating patient were subsequently assigned to the groups. The researcher assigning the intervention retained the random sequence.

### 2.5. Study Interventions

One group of participants received Aluneb Isotónico^®^ [Sakura Italia, Srl] medical device CE 0546 marked (Sodium hyaluronate: 0.1%, Xylitol: 5%, Potassium phosphate monobasic: 0.05%, Potassium phosphate dibasic: 0.03%, Water) while the control group received isotonic saline solution without HA and xylitol (Water, Sodium chloride: 0.9%, Potassium phosphate monobasic: 0.05%, Potassium phosphate dibasic: 0.03%). Both solutions were administered via the MAD Nasal™ [Aluneb, MAD Nasal Teleflex Medical, Ireland] medical device, a CE0120 marked and marketed Im medical device (nebulizer).

At the beginning of the study, all participants received training and instructions. They were shown how to use the device twice a day (morning and evening) following the procedures to introduce the medication or saline solution in the syringe and how to apply the liquid).

All patients began with the nebulization immediately that same day, in the evening after surgery. Patients were asked to perform nasal nebulization twice a day for 28 days.

All patients underwent endoscopic septoplasty with inferior turbinate submucosal resection, and no nasal packing was placed immediately after surgery. Septal Vicryl 4-0 “quilting” sutures were made through the septum to close any dead space and avoid hematoma collection. Endoscopic submucosa resection was performed using the Medtronic turbinate 11 cm × 2 mm resection blade (Medtronic Ref. 1882040) in all patients. 

### 2.6. Data Collection

In the baseline visit (pre-surgery or Visit 1), the following information was collected:

- Sinonasal medical assessment for diagnosis through recorded endoscopy and radiological evaluation (confirmation or disproval of a septal deviation, its severity, and the potential necessity for septoplasty with cauterization).
- Sino-Nasal-Outcome-Test survey of 22 indicators (SNOT-22), Visual Analogue Scale for nasal burning sensations, smell disturbances, taste disturbances, nasal bleeding, purulent rhinorrhea, headache, and sore throat (VAS main), and Nasal Obstructive Symptom Evaluation Scale (NOSE).
- Medical assessment by a detailed anamnesis (including potential concomitant treatments, if applicable).
- Review of their medical history
- Demographic data.

On the day of the surgery (Visit 2; day zero) and at Visits 3 (seven days), 4 ( fourteen days), and 5 (twenty-eight days), the following tests were performed: SNOT-22, VAS main, NOSE, Modified Lund-Kennedy endoscopic scale (MLK), The number of crusts and adhesions was recorded via equipment immediately before and after surgery as a baseline of the patient’s current status. 

The Spanish version of the Sino-Nasal Outcome Test 22 (SNOT-22) [21] and of the Nose Obstruction Symptom Evaluation (‘NOSE’) scale [22] were used for the subjective evaluation. The former is a questionnaire structurally composed of 22 related items that evaluate the severity of discomfort and symptoms that the patient has been experiencing over the past week due to chronic rhinosinusitis. Six visual analog scales (VASs) were used to measure the severity of symptoms (overall symptoms, nasal obstruction, headache, facial pain, smell disturbance, and nasal discharge). These were comprised of 100 mm lines with the extremes ‘no symptoms’ (0 mm) and ‘as bad as it can be’ (100 mm) [23]. The latter is a simple and fast questionnaire composed of five obstruction-related items that evaluate the severity of discomfort and symptoms that the patient has been experiencing over the past month due to nasal obstruction.

In addition, the nasal crusts and adhesions on both nostrils were counted and recorded with an endoscope. These were monitored by the surgeon and by an independent ENT who viewed the recordings following the procedures. Recording and analysis were performed on every visit.

The modified Lund-Kennedy endoscopic scale [24], was considered the most effective way to classify edema and discharge, with scoring from 0 to 6, as the Polyp score in the middle meatus was removed and considered 0. 

At the conclusion of the treatment phase (Visit 6, thirty-five days), subjects were contacted by telephone to monitor the product’s safety.

### 2.7. Primary Outcome

Our primary outcome was to evaluate the clinical efficacy and the quality of the post-operatory period in patients undergoing septoplasty with submucosal resection using Aluneb Isotónico^®^ as a post-surgical adjuvant treatment administered using the MAD Nasal™ medical device in comparison to isotonic saline solution.

### 2.8. Secondary Outcome

- Evaluation of the tolerability of the product
- Assessment of the clinical evolution post-surgery
- Evaluation of patients’ satisfaction with the treatment regarding their crust and adhesions.

Descriptive statistical analyses of the quantitative biometric variable results were performed at different experimental times, including basic descriptive parameters (central tendency and variation) that reliably define the distribution of the main variables at each time point. 

Baseline characteristics of the study participants were described with means and standard deviations for quantitative traits and percentages according to the group allocation.

Linear mixed-effects models were adjusted for data distribution of each response variable (MLK Oedema Scale, MLK Dischard Scale, SNOT-22 NOSE scale, and VAS scale) to assess the clinical response of the product (Aluneb Isotónico^®^) over the experimental times (days zero, seven, fourteen and twenty-eight). The effect of the product on the values of the main variables in the statistical analyses was interpreted in relation to the control group. In addition, model analyses include an investigation of the comparisons of each treatment at each time in relation to the baseline time.

Multiple biometric measurements over time (volunteers assessed at different time points), therefore correlated, were considered by including random effects for each individual, allowing the intercept of the models to vary at random between individuals in the study.

The significance of the effect of the product over time on the response variables was assessed using t-tests on the value of the estimated parameters in the linear mixed models. 

To address the potential effect of Aluneb Isotónico^®^ on the number of crusts over follow-up, we estimated the prevalence ratios assuming the presence of at least two crusts in both nostrils at each of the different visit times for the Aluneb Isotónico^®^ group compared to the control group.

The validity of each of the models was assessed via examination of the residuals.

Time was considered either a quantitative trait or a qualitative trait. 

The significance level was established at *p* < 0.05 for each statistical test performed in this study.

## 3. Results

At the beginning of our selection process, we included 58 patients (32 males and 26 females), randomly divided into:

- standard saline arm (group 1): 29 patients.
- standard saline plus HA and Xylitol arm (group 2): 29 patients.

At the end of the study, 49 patients continued and attended the follow-up period, and 9 were lost during the follow-up period. 

The control group was comprised of 22 patients (17 men, 5 women, 43 ± 14 years old), and the Aluneb Isotónico^®^ group was comprised of 27 patients (15 men, 12 women, 40 ± 17 years old) (Table 2). No statistically significant difference was found at baseline between the two groups, except for the weight of the subjects being lower in the Aluneb group. 

No complications were reported during surgery or during the follow-up period. Forty-nine patients attended the follow-up appointments. However, due to the COVID-19 pandemic, some patients, either because they were infected or were direct contact with infected individuals, could not attend the visits in person. Therefore, the endoscopy could not be performed. The evaluation of the subjective scales was carried out via telephone. In addition, some recordings of the endoscopies were lost due to technical difficulties. Table 3 therefore shows fewer patients, but it was nevertheless possible to formulate statistics using the results obtained. Nine patients were withdrawn from the study, three were lost due to screening failures and one was lost due to an adverse reaction requiring hospitalization, unrelated to the current research. All patients tolerated the nasal irrigation well, and none discontinued the treatment.

### 3.1. Objective Parameters Assessment

**Figure 1:**
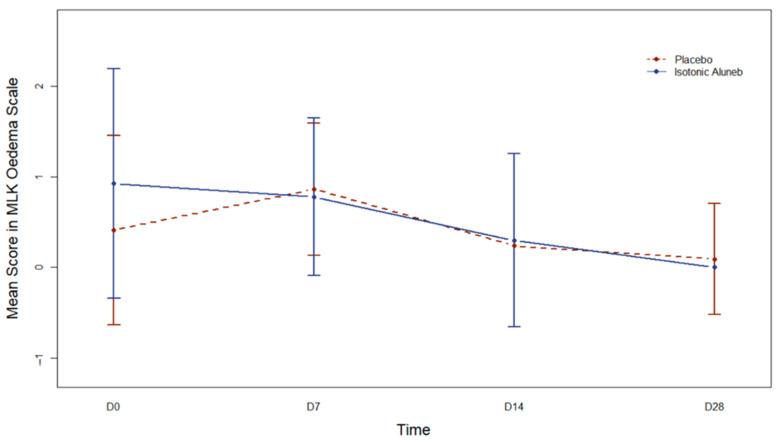
Mean (95% confidence interval) Modified Lund-Kennedy endoscopic scale (MLK) Oedema Scale along time according to the intervention group. D0: Day 0; D7: Day 7; D14: Day 14; D28: Day 28.

Regarding the MLK Oedema Scale (Figure 1), significant changes over time were observed in both intervention groups, with time as a continuous variable (*p* < 0.001 for both groups). Changes over time were also significantly different in both intervention groups when time was considered a continuous trait (*p* = 0.01). 

**Figure 2:**
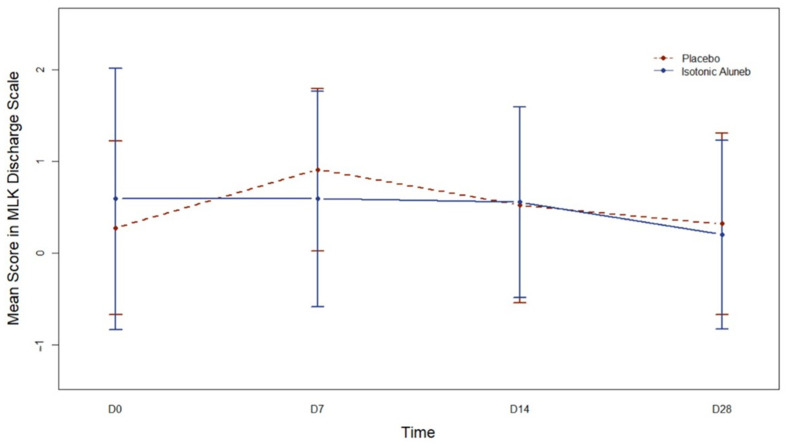
Mean (95% confidence interval) Modified Lund-Kennedy endoscopic scale (MLK) Discharge Scale along time according to the intervention group. D0: Day 0; D7: Day 7; D14: Day 14; D28: Day 28.

For the MLK Discharge Scale (Figure 2), significant changes over time were observed in the Aluneb Isotónico^®^ group with time as a continuous variable (*p* = 0.005). Models for the MLK Discharge Scale in the control group, including time as a continuous variable, did not converge. Nevertheless, with time as a qualitative variable, significant differences were observed on Day 7 compared to Day 0 (*p* < 0.001) but not on Days 14 or 28. Changes over time were not significantly different in both intervention groups (*p* = 0.17) when time was considered a continuous variable. However, when the time was considered a qualitative variable, changes over time were significantly different between both intervention groups at Days 7 (*p* = 0.01) and 28 (*p* = 0.03) but not at Day 14 (*p* = 0.21). 

Table 3 shows the risk ratio for two or more crusts in both nostrils. The presence of two or more crusts was significantly lower on Days 7 and 14. On Day 28, the percentage of participants with two or more crusts was lower in the Aluneb Isotónico^®^ group, although the comparison was not statistically significant. In other words, at one week and two weeks after surgery, there is a 55% and 49% reduction in the risk of developing two or more crusts in the Aluneb Isotónico^®^ group compared to the control group. Thus, Aluneb Isotónico^®^ is a protective agent against forming two or more crusts.

### 3.2. Subjective Parameters Assessment

All patients autonomously completed the SNOT-22, the NOSE, and the six VASs, at the three assessment times (pre-operatively and at D7, D14, and D28). However, the time required to complete the subjective self-assessment was at most 10 minutes.

**Figure 3:**
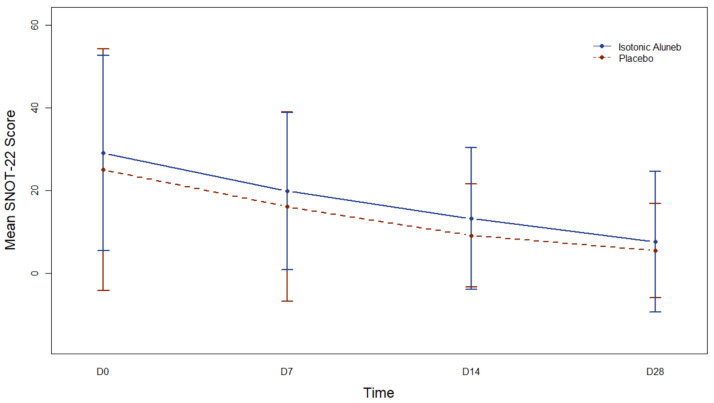
Mean (95% confidence interval) Sino-Nasal-Outcome-Test 22 (SNOT-22) Score along time according to the intervention group. D0: Day 0; D7: Day 7; D14: Day 14; D28: Day 28.

SNOT-22 scores decreased over follow-up in both groups (Figure 3). However, for the Aluneb Isotónico^®^ group and the control group, changes over time could not be assessed since neither the model with time as a quantitative trait or the model with time as a qualitative trait were adequate. Neither the model for the interaction between intervention groups and time with time as a continuous trait nor the model for the interaction between intervention groups and time with time as a qualitative trait to were able to address the association with SNOT-22 in an adequate manner.

**Figure 4:**
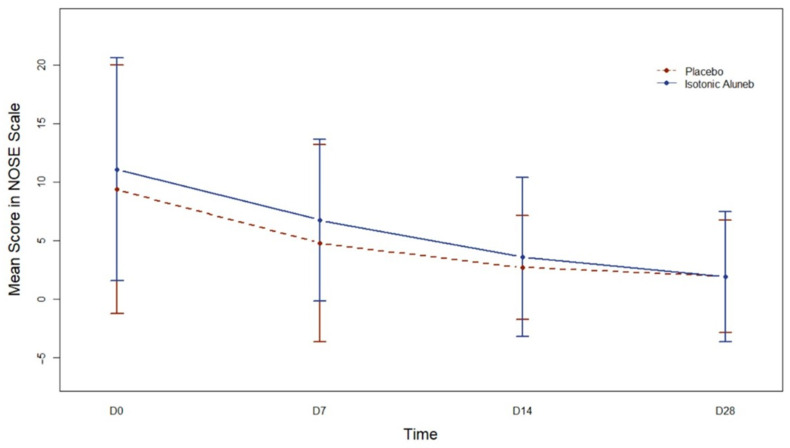
Mean (95% confidence interval) Nose Obstruction Symptom Evaluation (NOSE) Scale along time according to the intervention group. D0: Day 0; D7: Day 7; D14: Day 14; D28: Day 28.

Regarding the NOSE Scale (Figure 4), when time was considered a qualitative trait, all visits showed significantly different NOSE Scale values compared to the baseline visit (pD7 < 0.001, pD14 < 0.001, and pD28 < 0.001) in the control group. For the control group, models with time as a quantitative trait needed to be more adequate. In the Aluneb Isotónico^®^ group, changes over time were statistically significant when time was considered a continuous trait (*p* < 0.001). However, neither the model for the interaction between intervention groups and time with time as a constant trait nor the model for the interaction between intervention groups and time with time as a qualitative trait was adequate to address the association with the NOSE Scale.

**Figure 5:**
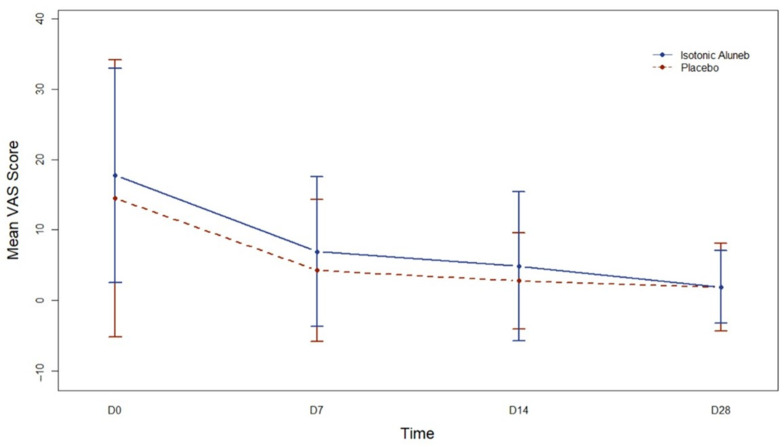
Mean (95% confidence interval) Visual Analogue Scale (VAS) Score along time according to the intervention group. D0: Day 0; D7: Day 7; D14: Day 14; D28: Day 28.

Main VAS Score (Figure 5) models for assessing changes over time needed to be more appropriate in the control group when time was considered a qualitative trait and when it was considered a quantitative trait. In the Aluneb Isotónico^®^ group, the model with time as a quantitative trait needed to be revised. However, significant differences were observed at all visits compared to the baseline visit in the Aluneb Isotónico^®^ group when time was considered a qualitative variable (pD7 < 0.001, pD14 < 0.001, and pD28 < 0.001). Residual analysis was not adequate, as neither the model for the interaction between intervention groups and time with time as a continuous trait nor the model for the interaction between intervention groups and time with time as a qualitative trait was able to address the association with Main VAS Score.

## 4. Discussion

This is the first study to be performed as a double-blind trial comparing Isotonic Saline HA with Xylitol given via low volume nebulizer with Isotonic saline solution in patients undergoing septoplasty and submucosa resection of inferior turbinates. The reported results show that the two products under investigation are well-tolerated and effective after surgery. 

Both treatments improved the quality of life of the patients for four weeks after the surgical procedure. The VAS scores significantly improved after each control point during the study in both groups, suggesting the efficacy of nasal irrigation of low volume after surgery. A similar result was obtained with the NOSE score and SNOT-22, both of which significantly improved in both groups after surgery. However, no significant differences were observed between the treatments. Nevertheless, the non-difference may be due to several reasons, among which it is essential to highlight the device used. The selection of MAD nasal for this study is linked to the commercial presentation of Aluneb Isotónico^®^, which recommends its use as a method of dispensing the solution. Therefore, to avoid breaking the randomization of the two groups, it was decided to use the same device. Including a third arm using a different device could have enriched the study and provided new evidence or significant differences in the parameters analyzed. In this study, we analyze the superiority of the solution, regardless the device used. 

According to a survey by Portela et al., nasal rinses are used in 93.2% of ENT follow-ups after nasal surgery [25]. Nevertheless, even though the data shows that there is an acceptance of the importance of some form of routine postoperative FESS care, more consensus is needed regarding precisely what the specific management routine should include. 

The EPOS 2020 guidelines for patients [26] has emphasized that the most effective nasal lavage is the most comfortable for the user. In this study, patients started using nasal nebulizer on the same day they underwent surgery. There were no complaints of unpleasant taste, smell, or difficulty performing the administration. This was most likely related to the device’s convenience and the low volume of solution delivery. We observed that the low-volume treatment was effective after surgery, although the results of the different quality-of-life scores were independent of the treatment. This observation aligns with what is described in the EPOS 2020 guidelines; no superiority between high-volume and low-volume delivery devices has been demonstrated.

After nasal surgery, mucosal damage is inevitable, which may lead to severe complications such as synechia and crusts. These can be due to several factors, such as chronic inflammation or secondary injury to remaining healthy tissues during the procedure, which can endanger the regeneration of the nasal mucosa [27]. The patient, consequently, shows an excess of secretion and edema in the 1st and 2nd week after surgery [28]. Regarding this, the study has demonstrated that in the first week after surgery, the Lund-Kennedy score increases significantly in the control group for edema and secretion. As described above, this would be the expected behavior after surgery. However, in the group using saline with HMW HA + Xylitol, there is no significant difference in edema and secretion in the first week compared to the start of treatment. This behavior is maintained for secretion in the second week and decreases significantly at four weeks, while edema reduces considerably at two and four weeks. Comparatively, between groups, the difference is significant at seven days regarding secretion.

Furthermore, the reduction in 55% and 49% risk of developing two or more crusts with the Aluneb Isotónico^®^ group compared to the control group at D7 and D14, respectively, was statistically significant, and is intimately related to the reduction of edema and secretion, clinically speaking, as during the first weeks after surgery, patients are particularly bothered by nasal congestion and discharge. This hypothesis agrees with the findings of other authors [29,30], who reported faster wound healing and re-epithelization in patients treated with a cross-linked hyaluronan gel post-FESS, which accelerates recovery, reduces pain, and promotes the regeneration of ciliated cells. It is known that the healing and repair of lesions is a complex process that restores the integrity and architecture of damaged tissue. It has been shown that HMW HA actively participates in several phases. Initially, it participates in the inflammatory response by promoting the recruitment of neutrophils through the activation of CD44 receptors. In a second instance, by promoting the proliferation of fibroblasts, which are the cells responsible for closing/repairing the lesion, through the deposition of collagen fibers, in addition to fostering re-epithelialization and recovery of nasal ciliated cells. The moisturizing effect of HA is also known to have a high water-binding capacity in its structure, which could confer an additional advantage for patients suffering from nasal crusts and atrophic rhinitis because it contributes to a better hydration of the mucous membrane. This presents an additional advantage for patients predisposed to post-surgical nasal crusting or atrophic rhinitis because it contributes to better hydration of the mucous membrane.

It is also important to mention the importance of the device and delivery methods to maximize local active ingredients in contact with the affected mucosa. According to the leaflet of MAD Nasal [31], it may nebulize in any position, and the nasal tip can be moved thanks to the malleable stylet. High plunger pressure guarantees that MAD will properly nebulize the liquid of the Aluneb Isotónico^®^ vial into the nasal cavity as a 30–100 m fine mist, and several studies demonstrate that this device nebulizer provided a more effective way to deliver local drugs to deeper and higher parts of the nasal cavity [32,33,34]. As in previous studies, we demonstrate the effectiveness of the MAD nasal in delivering an isotonic solution with HMW HA and Xylitol in the nasal mucosa and how this prevents crust formation and reduces nasal secretions in the first week after surgery compared to patients treated with isotonic solution only. In addition, some studies have shown that the use of MAD nasal covers more surface area and better distributes isotonic solution into nasal mucosa, resulting in greater bioavailability than other systems of delivery [35,36].

Avoiding crusting and edema will improve healing, allowing for less frequent visits to the office in the postoperative period and lower financial costs, allowing the surgeon to use their time in a more optimal way to see other patients. For the patient, this represents fewer follow-up visits to the physician´s office with faster and reliable improvement.

This study has several limitations. First, the number of enrolled patients is small, even if is it in line with previous studies in nasal surgery. For this reason, caution should be used when interpreting the results. However, the sample size was determined based on the main objective of the study and approved by the Ethics Committee. The calculated “n” was 58 patients (including a 20% dropout or withdrawal rate), with 29 in each treatment group. We managed to maintain a percentage of dropouts (9 in total) as expected and approved by the Ethics Committee.

Moreover, like the study by Macchi et al. [7], the control group of this study cannot be considered a placebo arm, as patients received the standard therapy of normal saline with the MAD nasal device. The possibility of detecting statistically significant differences between the groups might have been reduced due to this circumstance. However, including a control group of patients not undergoing nasal irrigation would be challenging to justify, as nasal irrigation is beneficial in the post-operative period [24,37]. Another potential fault of the study concerns the choice of primary outcome instrument. We decided to use the modified Lund Kennedy score for two reasons: first, it remains the most used endoscopic scoring system; second, it contains items that specifically address the post-surgical status of patients. Finally, the follow-up period was short (only four weeks); consequently, no information regarding the effect of nasal saline irrigation with or without sodium hyaluronate over a more extended period is available.

## 5. Conclusions

Although there is no current consensus regarding how to treat patients after nasal surgery, this study has shown that nasal washes with Aluneb Isotónico^®^ administered via MAD significantly improved nasal discharge and edema mainly during the first seven days after surgery and nasal symptoms (SNOT 22 and NOSE score) for all four weeks in the study compared to saline solution alone. It is a comfortable, easy, and safe treatment for patients. Furthermore, low-volume nasal nebulization via MAD nasal has been shown to improve patients’ quality of life after nasal surgery. However, further clinical studies with a more significant number of patients, with long-time follow-up, and compared against other devices for nasal irrigation are needed to confirm these preliminary findings.

## Figures and Tables

**Table 1:** Inclusion and exclusion criteria.

Inclusion Criteria
≥18 years of age.
Adequate level of understanding of the clinical study by the subjects and signing of the informed consent
Patients with moderate or severe septal deviation, with inferior turbinate hypertrophy, obstructing air passage through the nostrils.
Confirmation of the previous point by recorded endoscopy and radiological assessment by CT or ICAT facial structure.
Need for septoplasty with cautery, with or without the need for submucosal resection of inferior turbinates.
Minimum score of 25 on the SNOT-22 scale. Minimum mean score of 5 on the main VAS scale.
**Exclusion criteria**
<18 years of age
Patients treated with decongestants or antihistamines or having a significant medical need to receive them during their participation in the study.
Allergy, hypersensitivity, or any other type of incompatibility with any of the components of the product under study.
Pregnant or breastfeeding.
Patients participating in another clinical study or have experienced this in the last month.
Presence of other sinonasal disorders such as infection, epixtasis, coagulopathies, etc.
Diseases that require therapies that interfere with the evaluation of the product under study, especially drugs or medical devices for the postoperative period.
Patients that have surgery scheduled during their participation in the study or any other cause or aspect that, in the opinion of the researcher, may compromise compliance with the protocol (visits, adherence to treatment, compliance with questionnaires, etc.) or that does not make their participation in the study advisable.

**Table 2:** Baseline characteristics of the study participants.

	Aluneb Isotónico^®^ Group	Control Group	*p*-Value
N	27	22	
Age, years	40.30 (17.10)	43.45 (13.53)	0.47
Sex, % women	12, 44%	5, 23%	
Weight, kg	67.74 (16.81)	77.91 (13.17)	0.02
Height, cm	171.33 (9.66)	173.81 6.58)	0.30
Mean VAS score	17.74 (7.41)	14.50 (9.48)	0.20
Mean NOSE scale	11.07 (4.64)	9.36 (5.10)	0.23
Mean SNOT-22 test	29.07 (11.48)	25.05 (14.05)	0.29

Mean (standard deviation), unless otherwise stated.

**Table 3:** Prevalence ratio (95% confidence interval) for at least two crusts at the different visits in the Aluneb Isotónico^®^ group compared to the control group.

Crust All Patients						
**D0**	**Frequencies**		**Grade**		**Total**	**Risk ratio**
			**2 or +**	**0** **–** **1**		
	Treatment	Aluneb Isotónico^®^	2	24	26	1.62 (0.16, 16.61)
		Control group	1	20	21	
	**Total**		3	44	47	
**D7**	**Frequencies**		**Grade**		**Total**	**Risk ratio**
			**2 or +**	**0** **–** **1**		
	Treatment	Aluneb Isotónico^®^	8	17	25	0.45 (0.24, 0.84)
		Control group	15	6	21	
	**Total**		23	23	46	
**D14**	**Frequencies**		**Grade**		**Total**	**Risk ratio**
			**2 or +**	**0** **–** **1**		
	Treatment	Aluneb Isotónico^®^	8	16	24	0.51 (0.27, 0.98)
		Control group	13	7	20	
	**Total**		21	28	44	
**D28**	**Frequencies**		**Grade**		**Total**	**Risk ratio**
			**2 or +**	**0** **–** **1**		
	Treatment	Aluneb Isotónico^®^	2	16	18	0.24 (0.06, 1.01)
		Control group	6	7	13	
	**Total**		8	23	31	

## Data Availability

The data presented in this study are available on request from the corresponding author. The data are not publicly available due to originality of the work.
